# Supplementary material for: Protective Effects of Dietary Resveratrol against Chronic Low-Grade Inflammation Mediated through the Gut Microbiota in High-Fat Diet Mice
Source: Nutrients. 2022 May 10;14(10):1994. doi: 10.3390/nu14101994 (PMC9143590; doi:10.3390/nu14101994)
Supplement: Supplementary file 1 [file nutrients-14-01994-s001.zip › nutrients-1668995 - Updated Supplementary Material/nutrients-1668995.pptx]

## Slide 1
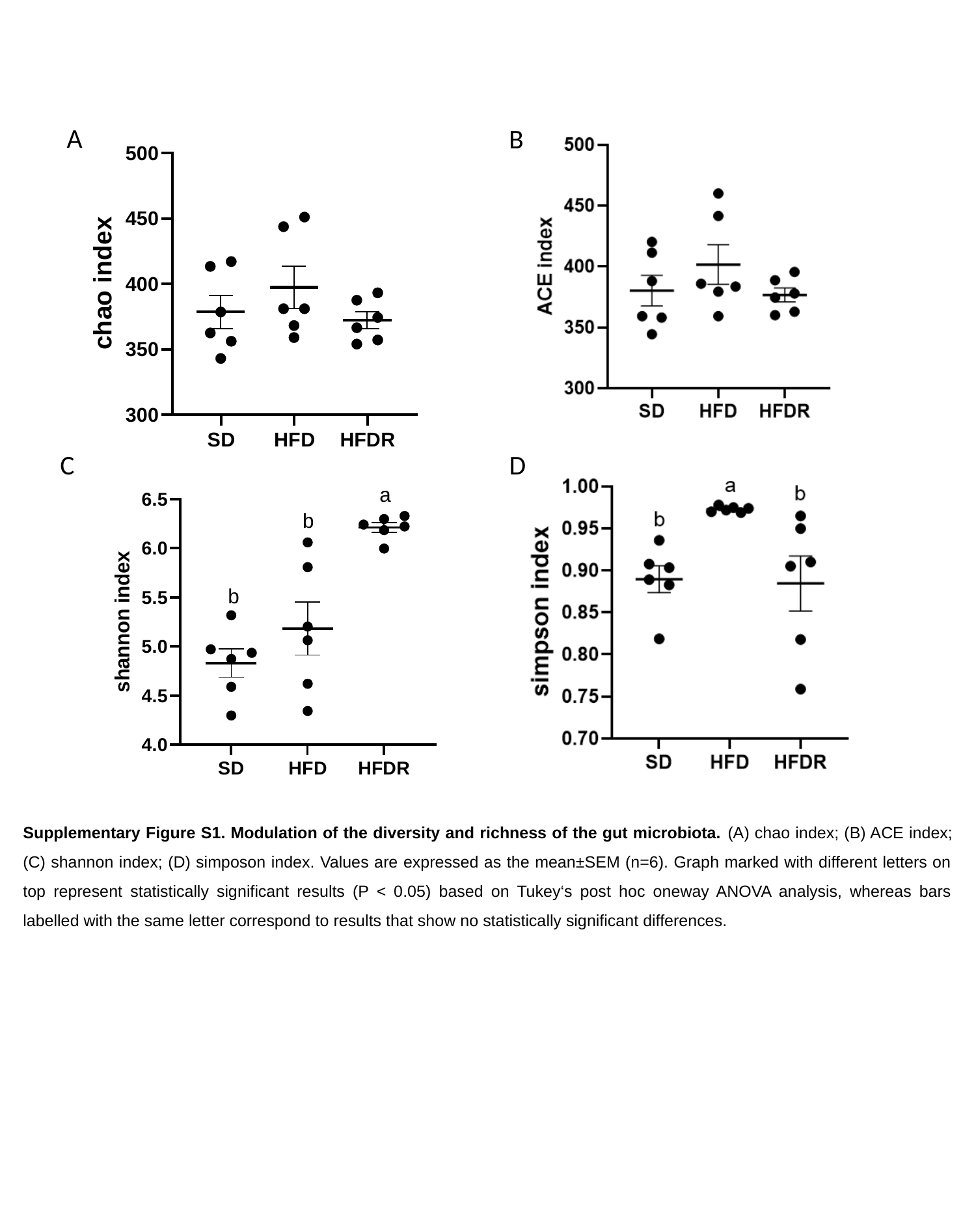

A
B
C
D
Supplementary Figure S1. Modulation of the diversity and richness of the gut microbiota. (A) chao index; (B) ACE index; (C) shannon index; (D) simposon index. Values are expressed as the mean±SEM (n=6). Graph marked with different letters on top represent statistically significant results (P < 0.05) based on Tukey‘s post hoc oneway ANOVA analysis, whereas bars labelled with the same letter correspond to results that show no statistically significant differences.

## Slide 2
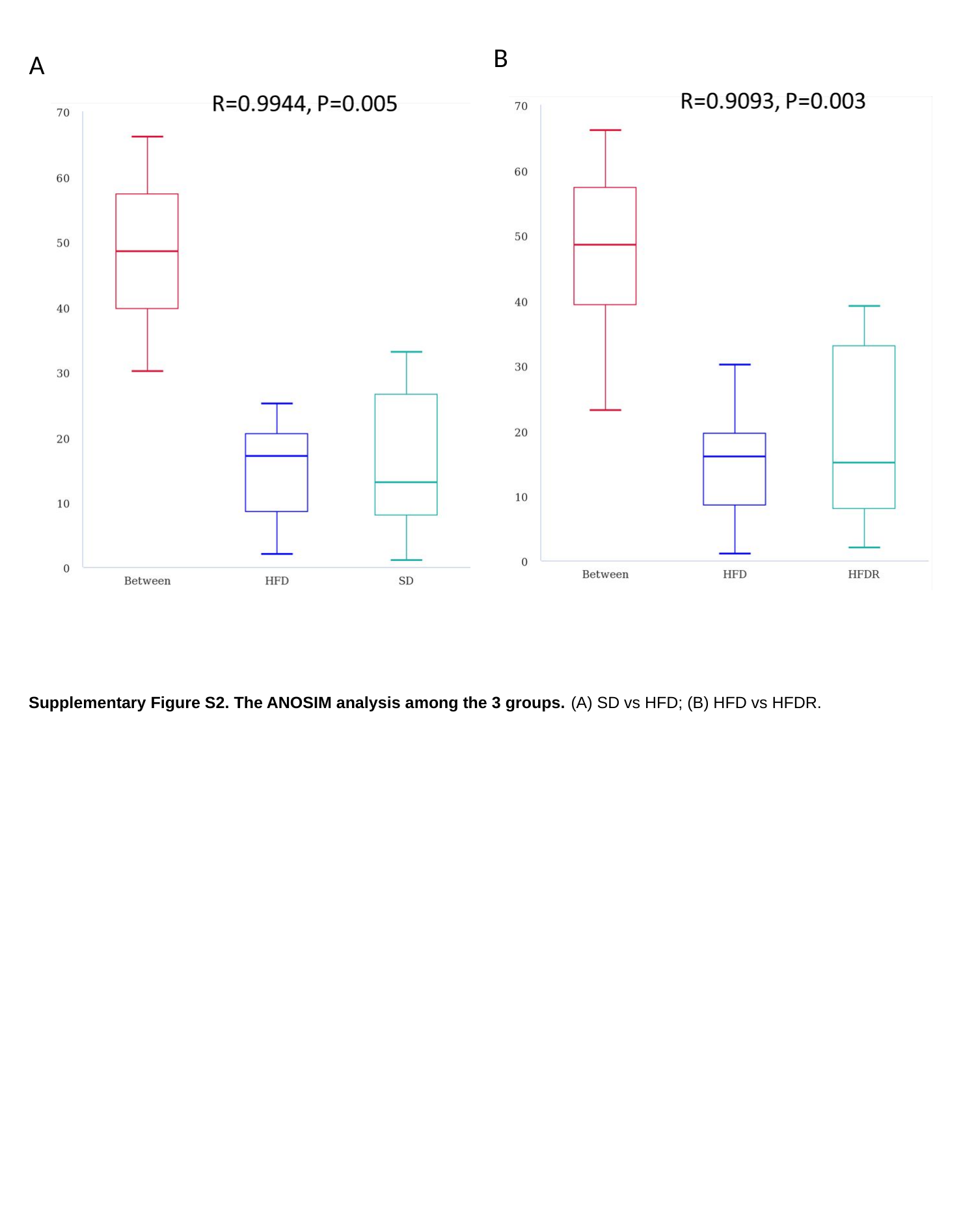

B
A
Supplementary Figure S2. The ANOSIM analysis among the 3 groups. (A) SD vs HFD; (B) HFD vs HFDR.

## Slide 3
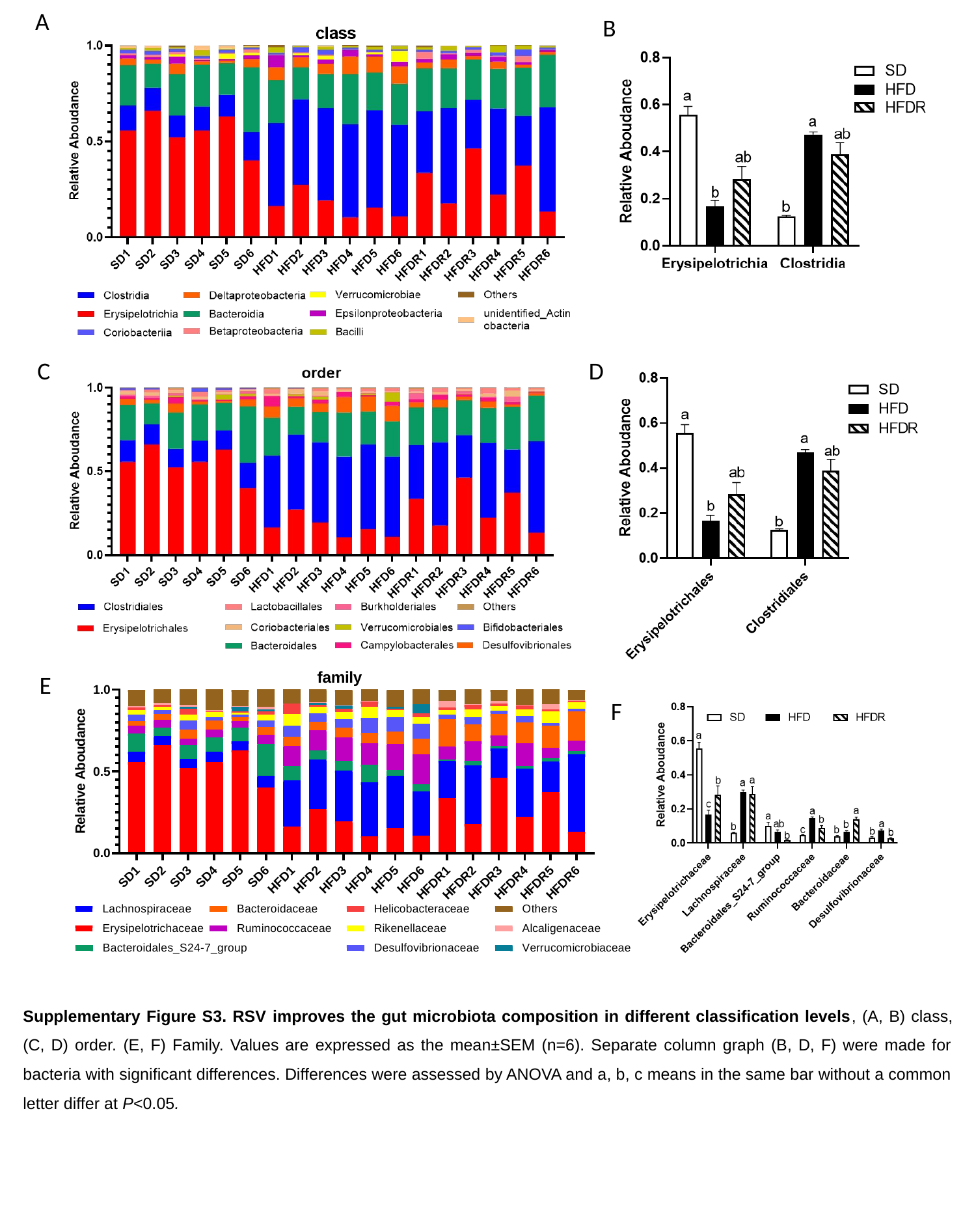

A
B
C
D
E
F
Supplementary Figure S3. RSV improves the gut microbiota composition in different classification levels, (A, B) class, (C, D) order. (E, F) Family. Values are expressed as the mean±SEM (n=6). Separate column graph (B, D, F) were made for bacteria with significant differences. Differences were assessed by ANOVA and a, b, c means in the same bar without a common letter differ at P<0.05.

## Slide 4
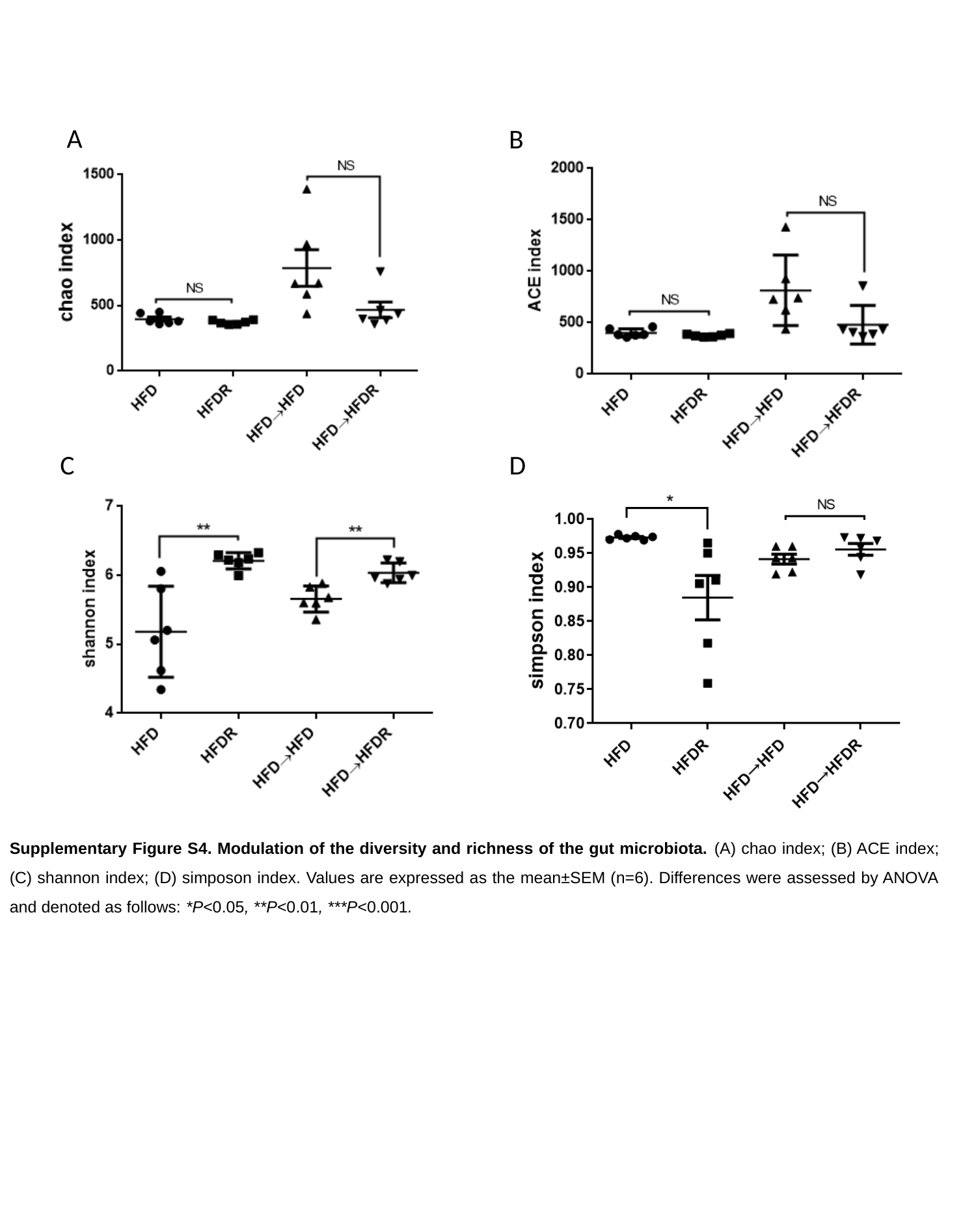

A
B
C
D
Supplementary Figure S4. Modulation of the diversity and richness of the gut microbiota. (A) chao index; (B) ACE index; (C) shannon index; (D) simposon index. Values are expressed as the mean±SEM (n=6). Differences were assessed by ANOVA and denoted as follows: *P<0.05, **P<0.01, ***P<0.001.

## Slide 5
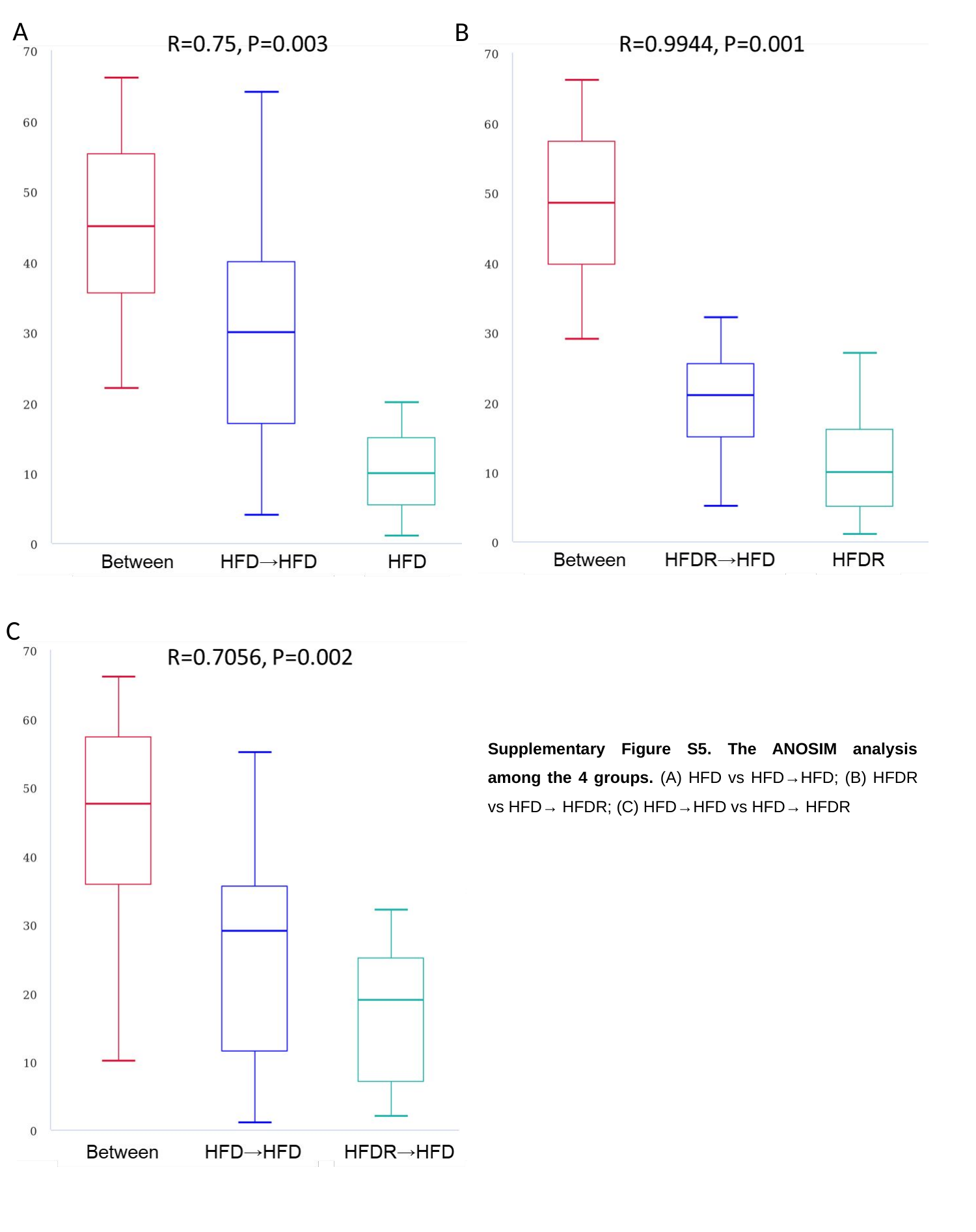

A
B
C
Supplementary Figure S5. The ANOSIM analysis among the 4 groups. (A) HFD vs HFD→HFD; (B) HFDR vs HFD→ HFDR; (C) HFD→HFD vs HFD→ HFDR
