# Supplementary material for: Protective Effects of Dietary Resveratrol against Chronic Low-Grade Inflammation Mediated through the Gut Microbiota in High-Fat Diet Mice
Source: Nutrients. 2022 May 10;14(10):1994. doi: 10.3390/nu14101994 (PMC9143590; doi:10.3390/nu14101994)
Supplement: Supplementary file 1 [file nutrients-14-01994-s001.zip › nutrients-1668995 - Updated Supplementary Material/supplement method.pdf]

Supplementary Methods

Gut microbiota analysis

Fecal samples from mice fed a SD, HFD or supplemented with RSV were collected, as well as HFD, HDR, HFD→HFD and HFDR→HFD group, snap-frozen and stored at -80 °C until use.

Extraction of genome DNA

Total DNA was extracted from fecal samples using CTAB/SDS method. DNA concentration and purity was monitored on 1% agarose gels. According to the concentration, DNA was diluted to 1 ng/μL using sterile water.

Amplicon Generation

16S/18S rRNA genes of distinct regions (16S V4/16S V3/16S V3-V4/16S V4-V5, 18S V4/18S V9, ITS1/ITS2, Arc V4) were amplified used the specific primer (e.g. 16S V4:515F-806R, 18S V4: 528F-706R, 18S V9: 1380F-1510R, ITS1: ITS5-1737F, ITS2-2043R, ITS2: ITS3-2024F, ITS4-2409R) with the barcode. All PCR reactions were carried out in 30μL reactions with 15μL of Phusion® High-Fidelity PCR Master Mix (New England Biolabs); 0.2μM of forward and reverse primers, and about 10 ng template DNA. Thermal cycling consisted of initial denaturation at 98 °C for 1 min, followed by 30 cycles of denaturation at 98 °C for 10 s, annealing at 50 °C for 30 s, and elongation at 72 °C for 30 s. Finally 72 °C for 5 min.

PCR Products quantification and qualification

Mix same volume of 1X loading buffer (contained SYB green) with PCR products and operate electrophoresis on 2% agarose gel for detection. Samples with bright main

strip between 400-450bp were chosen for further experiments.

PCR Products Mixing and Purification

PCR products was mixed in equidensity ratios. Then mixture PCR products was purified with GeneJET Gel Extraction Kit (Thermo Scientific).

Library preparation and sequencing

Sequencing libraries were generated using NEB Next® Ultra™ DNA Library Prep Kit for Illumina (NEB, USA) following manufacturer's recommendations and index codes were added.The library quality was assessed on the Qubit@ 2.0 Fluorometer (Thermo Scientific) and Agilent Bioanalyzer 2100 system. At last, the library was sequenced on an Illumina HiSeq platform and 250 bp paired-end reads were generated.

Data analysis

Paired-end reads from the original DNA fragments are merged by using FLASH (V1.2.7,http://ccb.jhu.edu/software/FLASH/), a very fast and accurate analysis tool which is designed to merge paired-end reads when there are overlaps between reads1 and reads2. Paired-end reads was assigned to each sample according to the unique barcodes. Sequences were analyzed using QIIME software package (Quantitative Insights Into Microbial Ecology), and in-house Perl scripts were used to analyze alpha- (within samples) and beta- (among samples) diversity. First, reads were filtered by QIIME quality filters. Then we use pick_de_novo_otus.py to pick operational taxonomic units (OTUs) by making OTU table. Sequences with ≥97% similarity were assigned to the same OTUs. We pick a representative sequences for

each OTU and use the RDP classifier to annotate taxonomic information for each representative sequence. In order to compute Alpha Divesity, we rarify the OTU table and calculate three metrics: Chao1 estimates the species abundance; Observed Species is estimates the amount of unique OTUs found in each sample, and Shannon index. Rarefaction curves were generated based on these three metrics.

QIIME calculates both weighted and unweighted unifrac, which are phylogenetic measures of beta diversity. We used Non-metric Multi-Dimensional Scaling (NMDS). NMDS help to get principal coordinates and visualize them from complex, multidimensional data. It takes a transformation from a distance matrix to a new set of orthogonal axes. By which the maximum variation factor is demonstrated by first principal coordinate, and the second maximum one by the second principal coordinate, and so on.
