# Supplementary material for: Protective Effects of Dietary Resveratrol against Chronic Low-Grade Inflammation Mediated through the Gut Microbiota in High-Fat Diet Mice
Source: Nutrients. 2022 May 10;14(10):1994. doi: 10.3390/nu14101994 (PMC9143590; doi:10.3390/nu14101994)
Supplement: Supplementary file 1 [file nutrients-14-01994-s001.zip › nutrients-1668995 - Updated Supplementary Material/supplement table total.pdf]

Table S1. The composition of experimental diets

Ingredient	Standard Diet		High-fat Diet	
	g%	kcal%	g%	kcal%
Casein, 30 Mesh	18.96	19.72	25.85	19.72
L-Cystine	0.28	0.30	0.39	0.30
Corn Starch	29.86	31.06	0.00	0.00
Maltodextrin 10	3.32	3.45	16.15	12.32
Sucrose	33.18	34.51	8.89	6.78
Cellulose, BW200	4.74	0	6.46	0.00
Soybean Oil	2.37	5.55	3.23	5.55
Lard	1.90	4.44	31.66	54.35
Mineral Mix S10026	0.95	0	1.29	0.00
DiCalcium Phosphate	1.23	0	1.68	0.00
Calcium Carbonate	0.52	0	0.71	0.00
Potassium Citrate, 1 H2O	1.56	0	2.13	0.00
Vitamin Mix V10001	0.95	1	1.29	0.99
Choline Bitartrate	0.19	0	0.26	0.00
Energy (kcal/g diet)	3.85		5.24	

Table S2. The sequences of primers used in Realtime PCR assays

Genes	Forward Primer	Reverse Primer
IL6	5'-CCGGAGAGGAGACTTCAC-3'	5'-TCCACGATTTCCCAGAG-3'
IL-1β	5'-TTGAAGAAGAGCCCATCCTC-3'	5'-CAGCTCATATGGGTCCGAC-3'
TNF-α	5'-TAGCCAGGAGGGAGAACAGA-3'	5'-TTTTCTGGAGGGAGATGTGG-3'
MCP-1	5'- TTAAAAACCTGGATCGGAACCAA-3'	5'- GCATTAGCTTCAGATTTACGGGT-3'

Table S3. Effect of RSV administration on obesity-related parameters

Data are expressed as the mean±S.E.M. (n=12). Statistical analysis was performed using ANOVA. The

Variables/Groups	SD	HFD	HFDR
Final body weight (g)	27.94±0.27^{b}	35.58±1.28^{a}	29.41±0.22^{b}
Weight gain (g)	6.13±0.19^{b}	14.49±1.09^{a}	8.18±0.34^{b}
Mean energy intake (Kcal/day/mouse)	10.86±0.15^{b}	14.07±0.27^{a}	14.09±0.27^{a}
Energy efficiency (g/Kcal)	0.42±0.01^{b}	0.49±0.01^{a}	0.52±0.01^{a}
Epi-WAT/body weight (%)	0.89±0.05^{c}	3.99±0.20^{a}	2.09±0.1^{b}
Per-WAT/body weight (%)	0.14±0.01^{c}	1.50±0.06^{a}	0.64±0.08^{b}
Ing-WAT/body weight (%)	0.52±0.05^{c}	1.49±0.07^{a}	0.92±0.07^{b}
BAT/body weight %)	0.45±0.08^{b}	0.44±0.02^{b}	0.83±0.05^{a}

means with different superscripts are considered significantly different (P<0.05), with the same letter correspond to results that show no statistically significant differences. Epi-WAT: epididymal white adipose tissue; Per-WAT:

perirenal white adipose tissue; Ing-WAT: Inguinal white adipose tissue; BAT: interscapular brown adipose tissue.

Table S4. Serum biochemical analyses from mice in SD, HFD and HFDR groups

	SD	HFD	HFDR
Fasting glucose (mmol/L)	$6.11±0.12^{b}$	$7.80±0.18^{a}$	$6.53±0.11^{b}$
Insulin (ng/mL)	$0.60±0.03^{b}$	$1.09±0.03^{a}$	$0.91±0.02^{c}$
Serum TC(mmol/L)	$2.74±0.07^{c}$	$4.18±0.07^{a}$	$3.53±0.05^{b}$
Serum TG(mmol/L)	$0.24±0.02^{b}$	$0.42±0.03^{a}$	$0.25±0.03^{b}$
Serum HDL(mmol/L)	$2.37±0.05^{c}$	$3.22±0.05^{a}$	$2.91±0.04^{b}$
Serum LDL(mmol/L)	$0.49±0.01^{c}$	$1.30±0.03^{a}$	$0.69±0.01^{b}$
ALT(U/L)	$25.68±1.08^{c}$	$46.69±1.40^{a}$	$32.75±1.74^{b}$
AST(U/L)	$105.88±7.01^{b}$	$132.25±4.59^{a}$	$111.00±2.94^{b}$

Data are expressed as the mean±S.E.M. (n=8). Statistical analysis was performed using ANOVA. The means with different superscripts are considered significantly different (P<0.05), with the same letter correspond to results that show no statistically significant differences.

Table S5. Effect of FMT administration on obesity-related parameters

Data are expressed as mean±S.E.M. (n=6). Differences were assessed by unpaired Student's t tests. The means with different superscripts are considered significantly different (P<0.05), with the same letter correspond to results that show no statistically significant differences. Epi-WAT: epididymal white adipose tissue; Per-WAT:

Variables/Groups	HFD→HFD	HFDR→HFD
Final body weight (g)	$35.24±0.37^{a}$	$31.26±0.47^{b}$
Weight gain (g)	$14.03±0.17^{a}$	$9.48±0.37^{b}$
Mean energy intake (Kcal/day/mouse)	$14.03±0.39^{a}$	$14.13±0.2^{a}$
Energy efficiency (g/Kcal)	$1.54±0.13^{a}$	$1.18±0.12^{a}$
Epi-WAT/body weight (%)	$3.74±0.48^{a}$	$1.98±0.15^{b}$
Per-WAT/body weight (%)	$1.58±0.24^{a}$	$1.03±0.07^{b}$
Ing-WAT/body weight (%)	$1.54±0.13^{a}$	$1.18±0.12^{b}$
BAT/body weight %)	$0.48±0.02^{b}$	$0.72±0.06^{a}$

perirenal white adipose tissue; Ing-WAT: Inguinal white adipose tissue; BAT: interscapular brown adipose tissue.

Table S6. Effect of FMT on serum biochemical parameters in C56BL/6 mice

	HFD→HFD	HFDR→HFD
Serum TC(mmol/L)	4.25±0.17[a]	3.91±0.27[b]
Serum TG(mmol/L)	0.81±0.11[a]	0.61±0.09[b]
Serum HDL(mmol/L)	3.11±0.33[a]	3.22±0.12[a]
Serum LDL(mmol/L)	1.38±0.14[a]	1.36±0.11[a]
TC/HDL	1.40	1.29
ALT(U/L)	67.00±5.76[a]	52.83±5.85[b]
AST(U/L)	139.17±10.22[a]	118.2±8.13[b]

Data are expressed as mean±S.E.M. (n=6). Differences were assessed by unpaired Student's t tests. The means with different superscripts are considered significantly different (P<0.05), with the same letter correspond to results that show no statistically significant differences.

Table S7. The Reads and OTU numbers of intestinal bacteria

Data are expressed as mean±S.E.M. (n=6). Statistical analysis was performed using ANOVA. The means

	Raw Reads	Qualified Reads	Average length	OTUs
SD	102605±2843[a]	84693±2424[a]	423±0.258 [a]	386.3±11.07 [a]
HFD	101393±1638[a]	84914±1440[a]	416.20±0.55[a]	399.60±7.24[a]
HFDR	99839±1848[a]	83285±1487[a]	417.40±0.85[a]	395.00±8.50[a]
HFD→HFD	98866±1611[a]	82974±1402[a]	417.10±0.53[a]	403.80±7.78[a]
HFDR→HFD	101717±1815 [a]	85382±1499[a]	415.10±0.80[a]	404.80±6.45[a]

with different superscripts are considered significantly different (P<0.05), with the same letter correspond to results that show no statistically significant differences.
